# Evaluation of tooth color change after a bleaching process with different lasers

**DOI:** 10.1007/s10266-023-00886-x

**Published:** 2024-02-17

**Authors:** Danny Möbius, Andreas Braun, Rene Franzen

**Affiliations:** 1 Department of Operative Dentistry, Periodontology and Preventive Dentistry, RWTH Aachen University, Pauwelsstraße 30, 52074 Aachen, Germany; 2 AALZ Aachen Dental Laser Center, Aachen, Germany

**Keywords:** Tooth bleaching, Diode laser, Color change, In-office bleaching, Spectrophotometer

## Abstract

The aim of this in vitro study was to evaluate the efficiency of diode laser-activated bleaching systems for color change of teeth. 75 extracted teeth were studied in five different bleaching protocols. Group 1: diode laser 445 nm, 320 µm fiber, 0.5W, continuous wave mode, *dose 53 J/cm*^*2*^*.* Group 2: diode laser 970 nm, 320 µm fiber, 1W, continuous wave mode*, dose 106.10 J/cm*^*2*^*.* Group 3: diode laser 940 nm, bleaching handpiece, 7W, continuous wave mode, *dose 105 J/cm*^*2*^*.* Group 4: diode laser 940 nm, 300 µm fiber, 2W, continuous wave mode, dose *47.16 J/cm*^*2*^*.* Group 5: bleaching process without laser activation. In groups 1, 2 and 5, teeth were bleached with Perfect Bleach Office + and in groups 3 and 4, LaserWhite20 bleaching gel was used. Tooth color was determined immediately after the bleaching process using a spectrophotometer. Color change data on the CIE *L* * *a* * b* system was analyzed statistically by the one-way ANOVA and Tukey’s HSD test. All bleaching procedures resulted in a change of color. All laser groups (∆E * *ab* > 3) have statistically larger ∆*E* * *ab* values than the control group (∆*E* * *ab* = 0.73) (*p* < 0.05). The diode laser 445 nm has the largest ∆*E* * *ab* value (∆*E* * *ab* = 4.65) and results in a significantly higher color difference than all other groups. In terms of color score difference in VITA Shades, all laser-activated groups lead to a lightening effect while the control group leads to only a slight lightening effect. The diode laser 445 nm produced the greatest color difference. Laser-activated bleaching is more effective than conventional bleaching without light activation. The diode laser 445 nm performs best in this in vitro study.

## Introduction

Esthetics plays an important role in dentistry. In addition to the size, shape and position of the teeth, the color is also a decisive factor [1, 2]. A light tooth color is seen by patients as an expression of health, aesthetics and attractiveness [3]. Modern tooth whitening procedures provide the basis for quick and successful results in this respect [4, 5]. Professional bleaching of discolored teeth was first described by M'Quillen in 1867 [6]. Electromagnetic irradiation was first used in 1937 to increase the effectiveness of bleaching [7]. In the 1990s, a bleaching gel was introduced that allowed the application of bleaching agents at home and established the popular technique of home bleaching [8–10]. “Home bleaching is the most commonly used method. Home treatment is often performed with carbamide peroxide concentrations ranging from 10 to 16%; in practice, approximately 35–37% carbamide peroxide is used. However, according to studies, both methods produce similar color improvement after treatment is completed [11]. However, even though home bleaching is effective, there are some obvious advantages to in-office bleaching at the dentist's office: These include control by the dentist, the avoidance of soft tissue damage, the reduction in overall treatment time and potential for immediate results to patient satisfaction [4].

Thus, one of the more modern tooth whitening procedures is laser-assisted in-office bleaching, in which the traditional energy sources (such as heat or light) needed to activate the bleaching gel are replaced by a laser system [12–16]. Laser tooth bleaching first began in 1996 with the approval of the argon laser (488 nm) and the CO_2_ laser (10,600 nm) [5]. In the method, the bleaching gel is applied to the teeth by the dentist and activated with laser light. This heats the gel and releases more radicals in the gel, which oxidize the color pigments and make the teeth look whiter [17]. However, there is an additional risk that the pulp may also be heated and suffer irreversible damage as a result. This must be considered when bleaching vital teeth [18]. In addition, numerous studies have described damage to the tooth structure caused by bleaching processes. Hydrogen peroxide has been found to be able to dissolve minerals from enamel [19–21]. Shortening the total treatment time results in less contact time of the gel with the tooth surface and thus has a preventive effect against tooth structure changes [22].

In-office bleaching is the fastest option of bleaching, which is also investigated in this study. Different lasers can be used for this procedure, which then differ in the duration and effectiveness of whitening [12, 23–25]. A wide variety of lasers have been studied, including, for example, the KTP laser, the Er:YAG laser, and all diode lasers in a wide variety of wavelengths [12, 26]. The Food and Drug Administration has officially approved the argon ion laser, CO_2_ laser and diode laser for dental applications [27].

445 nm wavelength is still quite new in current studies and only described in a few studies [28–30]. Recently, the first studies on the diode laser with a wavelength of 445 nm for bleaching can be found [31, 32]; however, only the microhardness and temperature of the pulp are considered here and not the color change. The effectiveness of bleaching is often determined by comparing tooth color with tooth shade guides. Given the shortcomings of this method and its subjective nature, a spectrophotometer is usually used for this purpose to minimize the effects of environmental factors on color perception.

The purpose of this study is to evaluate the effectiveness of different lasers and bleaching gels and to document the changes in color coordinates immediately after the bleaching process.

In this study, the effectiveness of laser bleaching with three different wavelengths (445 nm, 940 nm, 970 nm) of diode lasers was investigated in comparison to conventional bleaching.

The working hypothesis is: laser-activated bleaching with the diode laser 445 nm, 940 nm and 970 nm results in a better bleaching outcome than conventional bleaching without light activation.

## Materials and methods

### Preparation of the samples

In this study, 75 caries-free and filling-free extracted teeth with intact enamel surfaces were used. The teeth were extracted by dentists and oral surgeons in the Aachen, Düsseldorf, and Dortmund region due to non-preservability. Teeth were cleaned with scalers and polishing brushes using a non-fluoride pumice paste and stored in a uniform solution (NaCl 0.9% and NaN_3_ 0.001%) to disinfect them. Subsequently, all teeth were transferred to distilled water to avoid subsequent interactions of the bleaching gel with the rinsing solution. The distilled water was changed weekly to prevent bacterial growth.

All teeth were shortened at the root by 2 mm with a water-cooled diamond saw. The root canals were visited and visualized with K-files ISO 15–20 from apical. This was followed by mechanical widening with diamond drills and Gates Glidden drills. Finally, the canals were checked for mobility with K-files ISO 25 and prepared so that the cannulas could be inserted via the apex for blood flow simulation. Oral access for the thermal probes was drilled with diamond burs.

The teeth were rinsed with NaOCl and EDTA to remove both organic and inorganic components in the pulp cavum.

After the teeth were cleaned and prepared, they were conditioned at the apex for bonding with composite using the cannulas. Five teeth were randomly grouped into a quadrant block consisting of a central incisor, a lateral incisor, a canine, a first premolar, and a second premolar. The teeth, together with the inserted cannulas, were positioned in a plastic and rubber dam casting mold and embedded in a polyurethane casting resin, with a similar thermal capacity to human bone [33, 34]. Bone has a thermal conductivity of 0.58–1.2 W∕(m K), a density of 1.36 kg∕cm^3^ and a heat capacity of 1.6–2.1 kJ∕(kg K) [34]. In this respect, polyurethane casting resin was the most suitable material [33].

### Blood flow simulation

**Fig. 1:**
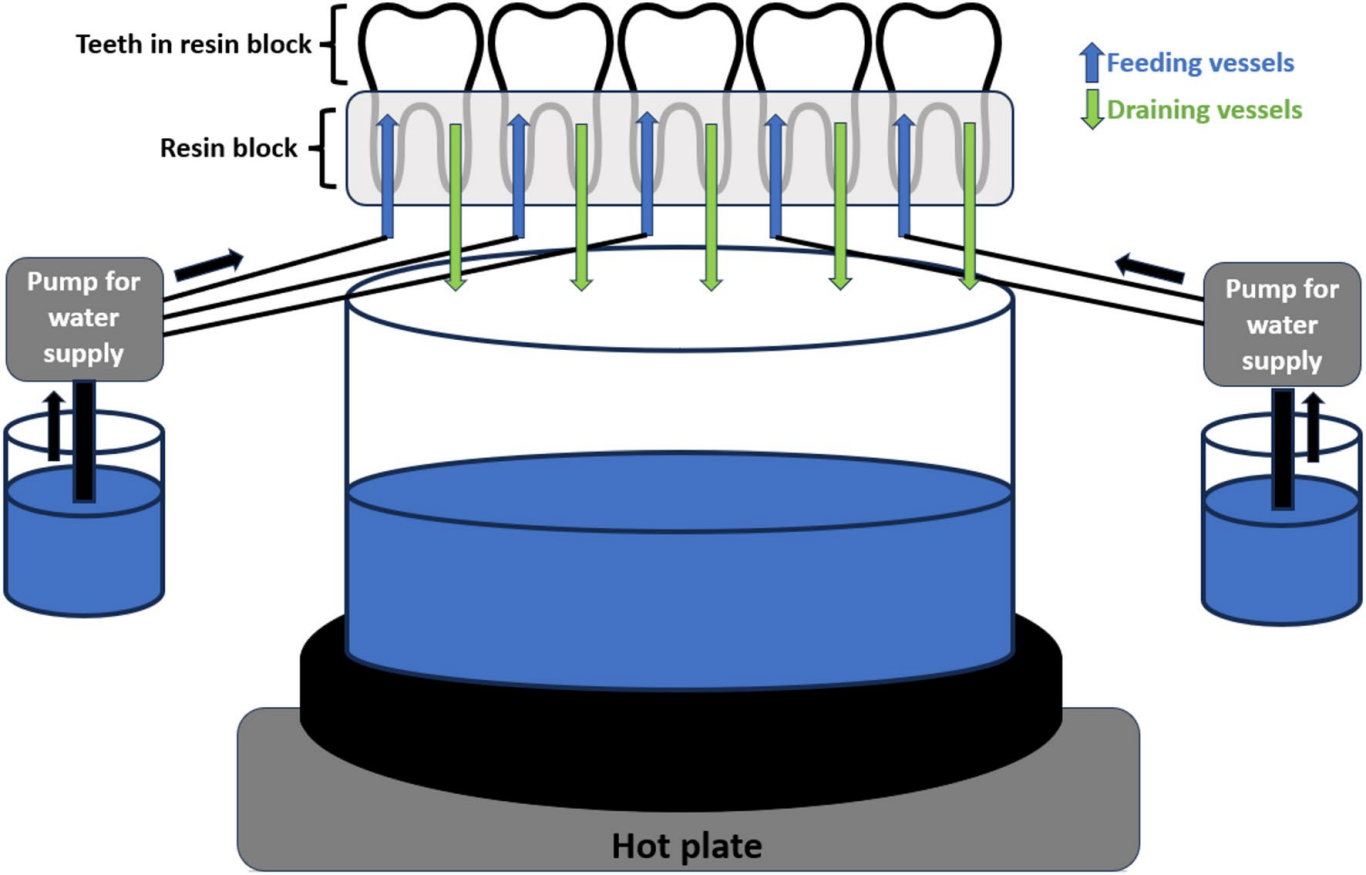
Illustration of the blood flow simulation

In this study, the physiological blood flow was simulated approximately. Depending on the weight of the tooth and the tooth pulp, a physiological blood flow of 2–6.6 µl/min is approximately obtained [35]. Teeth were prepared at the root so that two cannulas Capillary Tips 0.48 mm (Ultradent Products GmbH, Germany) could be inserted and sealed per tooth using composite. One cannula was used for water inflow and the other cannula was used for water outflow. Water was continuously pumped through the dental pulp with peristaltic pumps from Ismatec (Pole-Carmer GmbH Wertheim, Germany) at a flow rate of 5.93 µl/min. This value was the lowest flow achievable by these pumps and well within the approximate physiological conditions in the context of this study. In addition to the flow velocity, the temperature of the blood is decisive, which in humans is at a body temperature of approx. 38 °C [36]. The water supplied was therefore additionally heated by a heating plate so that a water temperature of 36–38 °C approximately prevailed in the dental pulp. Figure 1 shows a sketch of the blood flow simulation.

### Color measurement

The color of the teeth was always determined by the same person using the Shadepilot spectrophotometer (DeguDent GmbH, Germany) based on the CIE *L* * *a* * b* color system. In the CIE *L* * *a* * b* color system, the L* axis represents the value brightness (or darkness), the a* axis is a measure of redness (positive a*) or greenness (negative a*), and the b* axis is a measure of yellowness (positive b*) or blueness (negative b*). The difference between the color coordinates was calculated as follows:

$$\Delta E*_{ab} = \sqrt {{(\Delta L*)^2 + \left( {\Delta a*} \right)^2 + (\Delta b*)^2 }} .$$

Delta *E* is a measure of perceived color distance and was used in this study to quantify the brightening effect. In addition to the CIE L * *a* * b* color system, the spectrophotometer calculates the appropriate VITA classical A1–D4 shade based on the lowest Delta *E* value, which served here for visualization and is more practical. All measurements were taken under the same lighting conditions behind a darkening curtain. A black card was positioned directly behind the tooth to avoid possible light reflections. The spectrophotometer has a native white balance by first photographing a white tile and then a green tile. Seven measurements were taken for each tooth and the mean values were calculated. Each measurement focused on the center of the tooth. After the photographs were taken with the spectrophotometer, the calculated VITA Shades were matched with the Nikon D7000 (Nikon, Germany) and the VITA classical shade tabs (VITA Zahnfabrik, H. Rauter GmbH & Co. KG). The digital photographs were taken using an 18% gray card for automatic white balance and thus equal exposures of all photographs [37]. The VITA Shades were also sorted by brightness level according to the following scheme (Table 1).

**Table 1:** Shade ranging from 1 to 16 (brightest to darkest) according to VITA classical shade guide

Vitashade	B1	A1	B2	D2	A2	C1	C2	D4	A3	D3	B3	A3,5	B4	C3	A4	C4
Score	1	2	3	4	5	6	7	8	9	10	11	12	13	14	15	16

### Bleaching procedure

Two different bleaching gels were used for laser bleaching, each matched to their respective laser. Perfect Bleach Office + (Voco GmbH) was used together with diode laser 445 nm (Dentsply Sirona, USA) diode laser 970 nm (Dentsply Sirona, USA) and as a control group without light activation (groups 1, 2 and 5). The base and activator were mixed automatically while operating the syringe to form a gel with a hydrogen peroxide concentration of 35%.

The second gel is LaserWhite20 (Biolase, USA), which was used with the diode laser 940 nm (Biolase, USA), both with the bleaching handpiece and with the fiber (groups 3 and 4). This gel was previously mixed from the base gel (45% hydrogen peroxide concentrate) and the activator to form a 35%gen bleaching gel. The bleaching gel was then evenly applied to the vestibular surface in a layer 1–2 mm thick, irradiated with the appropriate laser depending on the group, and finally rinsed with water.

The bleaching process took place in five different groups as follows:

*Group 1*: diode laser 445 nm, 320 µm fiber, 0.5 W, continuous wave, irradiation time 30 s, beam radius 0.3 cm (distance 2 cm) —>  dose 53.05 J/cm^2^.

After Perfect Bleach Office + bleaching gel (Voco GmbH) was applied to all five teeth of the quadrant, it was immediately irradiated with the laser for 30 s each. After all five teeth had been irradiated, the activated gel remained on the teeth for a further 15 min and was finally rinsed off with water. Total gel application time of 17 min and 30 s.

*Group 2*: diode laser 970 nm, 320 µm fiber, 1 W, continuous wave, irradiation time 30 s, beam radius 0.3 cm (distance 2 cm) —> dose 106.10 J/cm^2^.

The bleaching procedure was performed in the same way as in Group 1.

*Group 3*: diode laser 940 nm, bleaching handpiece, 7 W, continuous wave, 1–2 mm distance, irradiation time 60 s, beam area: $4\,{\text{cm}}*1\,{\text{cm}} = \left( {4\,{\text{cm}}} \right)^2$ — > dose 105 J/cm^2^.

After the LaserWhite20 bleaching gel (Biolase, USA) was mixed and applied to all five teeth of the quadrant, the quadrant as a whole was irradiated with the bleaching handpiece for 30 s according to the manufacturer's instructions. This was followed by a pause of 1 min and 30 s, then the quadrant was irradiated a second time for 30 s. The gel was left on the teeth for 15 min and then rinsed with water. Total gel application time of 17 min and 30 s.

*Group 4*: diode laser 940 nm, 300 µm fiber, 2W, continuous wave, irradiation time 60 s, beam radius 0.9 cm, (2 cm distance) —> dose 47.16 J/cm^2^.

After LaserWhite20 bleaching gel (Biolase, USA) was applied to all five teeth of the quadrant, each tooth was irradiated with the laser for 30 s. This procedure was immediately repeated a second time for all five teeth and the gel was left for 15 min with a final rinse with water. Total gel application time of 20 min.

*Group 5*: bleaching process without laser activation.

Perfect Bleach Office + bleaching gel (Voco GmbH) was applied to the teeth in a 1–2 mm layer according to the manufacturer's instructions and left there for 15 min. The gel was then rinsed off under running water.

**Fig. 2:**
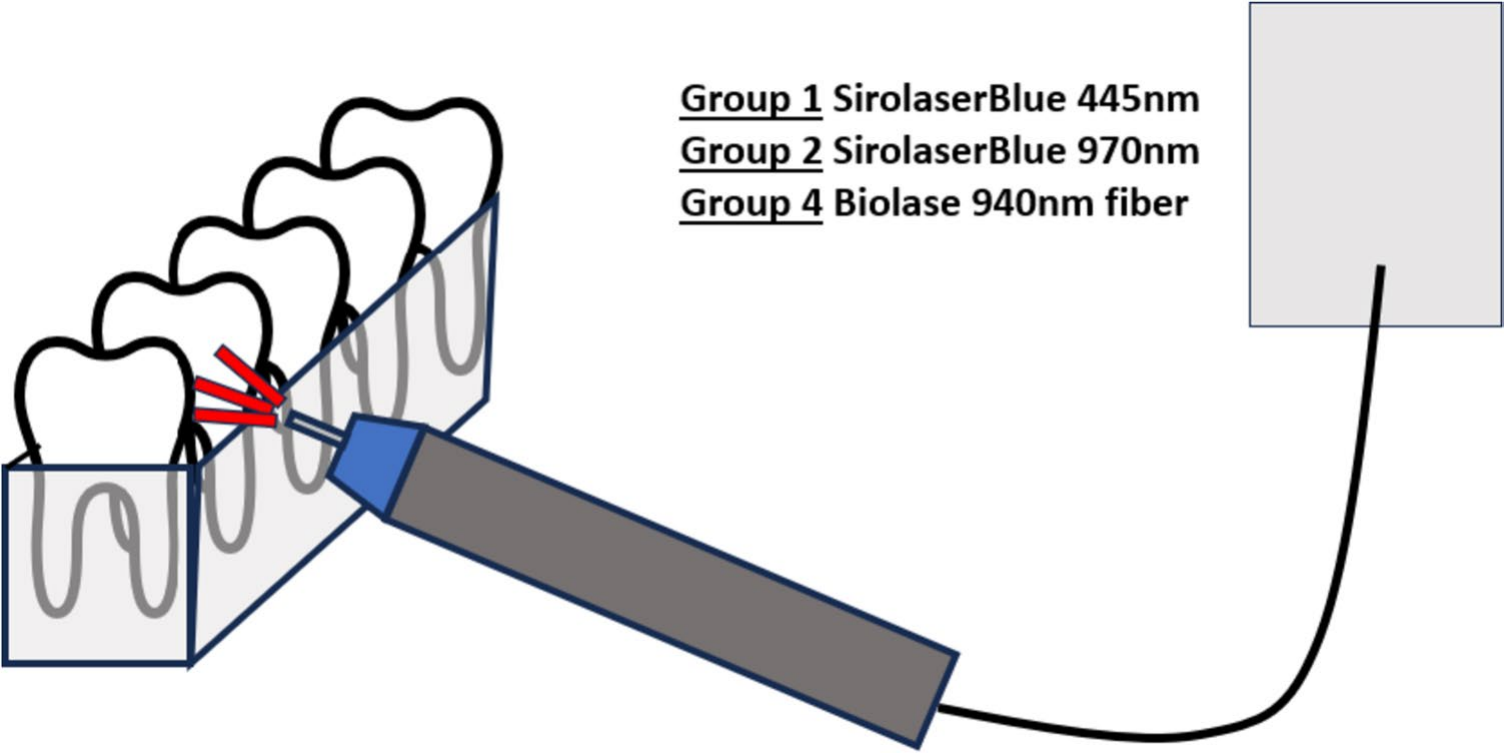
Illustration of the bleaching process with the laser fiber. All five teeth are irradiated one after the other

**Fig. 3:**
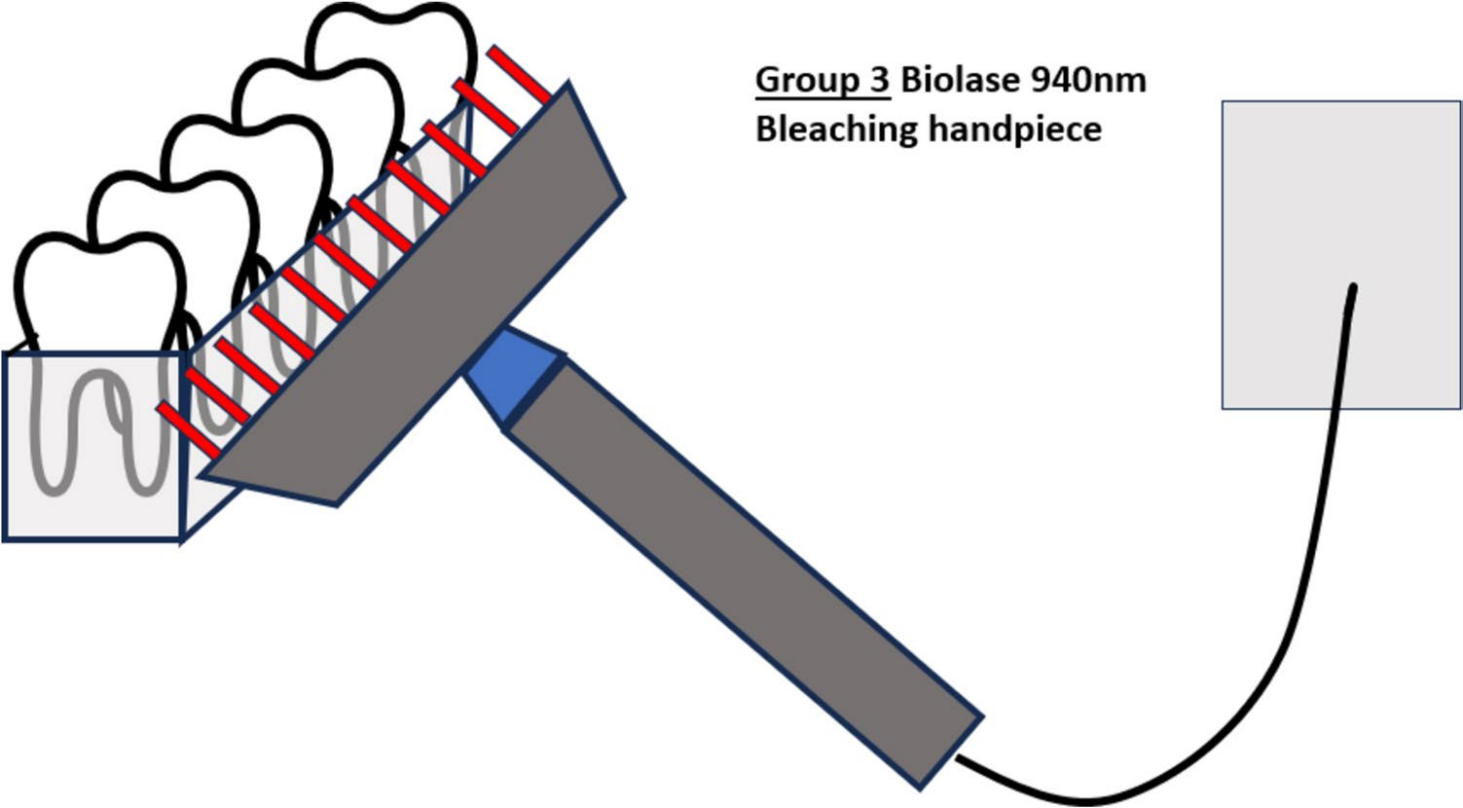
Illustration of the bleaching process with the bleaching handpiece. All five teeth are irradiated at the same time

The color was measured immediately after the bleaching process. Figures 2 and 3 show the various bleaching processes in outline form.

### Statistical analysis

The data were analyzed with the software program IBM SPSS Statistics.

A factorial analysis of variance was performed between the five groups and within groups, as well as a post hoc Tukey test. The significance level was set at *p* value = 0.05.

## Results

All 75 teeth were evaluated regarding their color difference individually and independently of their constellation in a block of five. Mean values were calculated from seven measurement repetitions per tooth. These contain the coordinates CIE *L* * *a* * b* and the resulting tooth shade Vitapan^®^ classical shade system. The tooth color of the Vitapan classical was divided into 16 different color scores according to its brightness.

The differences of the color coordinates and the color distance ∆*E* * *ab* from the CIE *L* * *a* * b* color space was determined from the mean values. The color distance ∆*E* * *ab* is calculated according to the formula:

$$\Delta E*_{ab} = \sqrt {{(\Delta L*)^2 + \left( {\Delta a*} \right)^2 + (\Delta b*)^2 }} .$$

The mean values and standard deviations of the tooth color parameters for each group are shown in Table 2 (∆*E* * *ab*, ∆*L*, ∆*a*, ∆*b*, color score difference).

**Table 2:** The mean tooth color change parameters and standard deviations (∆*E* * *ab*, ∆*L*, ∆*a*, ∆*b*, color score differences in VITA Shade)

Group		∆E*ab	∆*L*	∆*a*	∆*b*	Color score differences in VITA Shade
Group 1	Mean	4.6	3.1	− 0.3	− 3.2	4.2
	*SD*	1.9	1.1	0.4	1.7	2.1
Group 2	Mean	3.1	2.6	0.0	− 1.4	1.8
	*SD*	1.0	0.7	0.3	1.2	2.5
Group 3	Mean	3.1	2.2	0.0	− 1.9	3.4
	*SD*	1.4	1.5	0.3	0.9	2.2
Group 4	Mean	3.2	2.0	0.0	− 2.1	1.3
	*SD*	1.9	1.0	0.3	2.0	2.2
Group 5	Mean	0.7	0.2	0.1	− 0.2	− 0.3
	*SD*	0.4	0.5	0.2	0.5	2.1
Total	Mean	3.0	2.0	0.0	− 1.8	2.1
	*SD*	1.9	1.4	0.3	1.7	2.7

All groups show an increase in brightness (*L*-parameter) and a decrease in yellowness (*b*-parameter). The *a*-parameter (redness or greenness) hardly changes, and the groups do not differ significantly. Teeth whitened by laser activation (groups 1–4) are significantly brighter than teeth whitened only conventionally without laser (group 5) (*p* < 0.05). There was no significant difference between the laser groups. The diode laser 445 nm results in the greatest reduction in yellowness followed by the diode laser 940 nm (with bleaching handpiece). These two groups were significantly different from the control group (*p* < 0.05).

In terms of ∆*E* * *ab* value, all groups were effective in bleaching the teeth. The group 1 produced the greatest color difference with a value of 4.6. The other three laser groups (groups 2–4) differed only minimally with values of 3.1–3.2. The control group (group 5) produced the least color difference with ∆*E* * *ab* = 0.7. Group 1 has the largest ∆*E* * *ab* value and results in a significantly higher color difference than all other groups (*p* < 0.05).

The other laser groups (2–4) do not differ significantly among themselves with respect to the whitening effect (*p* > 0.05).

All laser groups have significantly larger ∆E * *ab* values than the control group (*p* < 0.05).

In terms of color score difference in VITA Shades, all laser-activated groups lead to a lightening effect, while the control group remains almost unchanged. These results also support the results of the ∆*E* * *ab* values since the diode laser 445 nm also produced the greatest color difference (4.2 VITA Shades). In this case, however, the bleaching handpiece stands out from the other two laser groups with a value of 3.4 VITA Shades. The diode laser 970 nm (1.8 VITA Shades) and the diode laser 940 nm with fiber (1.33 VITA Shades) resulted in comparable color changes.

The diode laser 445 nm is significantly more effective than the diode laser 970 nm, diode laser 940 nm with fiber and the control group. The diode laser 940 nm with the bleaching handpiece results in the second highest color lightening with respect to the VITA classical color scale. This is significantly better than the control group. However, the other experimental groups (groups 2, 4, 5) do not differ significantly (*p* > 0.05).

In Tables 3 and 4, the laser groups (groups 1–4) are compared with the control group (group 5) in terms of ∆*E* * *ab* and color score differences in VITA Shades.

**Table 3:** Laser groups versus control group compared in terms of ∆*E* * *ab*

Group	Mean ∆*E* * *ab*	*SD*
Laser groups 1–4	3.5	1.7
Group 5	0.7	0.4
Total	3.0	1.9

**Table 4:** Laser groups versus control group compared in terms of color score difference in VITA Shades

Group	Mean color score differences in VITA Shade	SD
Laser groups 1–4	2.7	2.5
Group 5	− 0.3	2.1
Total	2.1	2.7

Teeth whitened with the laser-activated bleaching method lightened by an average of ∆*E* * *ab* = 3.5 and teeth whitened without light activation have a color difference of ∆*E* * *ab* = 0.7.

The teeth of the laser groups become brighter by an average of 2.7 VITA Shades. In contrast, the teeth of the control group experience almost no color change (− 0.3 VITA Shades).

The laser groups differ significantly from the control group regarding to both parameters.

## Discussion

This in vitro study shows the effectiveness of whitening different diode laser systems compared to power bleaching without light activation. The diode laser 445 nm, diode laser 970 nm and diode laser 940 nm (bleaching handpiece and fiber) were investigated.

The initial color of the teeth also affects the whitening result. The lighter a tooth at the beginning, the lower is the whitening effect and, conversely, the more yellow or dark a tooth, the stronger is the expected whitening effect [38]. However, the teeth were randomly distributed to all five groups here, so that the baseline prerequisites were randomized.

Since visual tooth color determination is very subjective and depends on all factors such as different lighting conditions or the anatomy of the observer, color measurement is performed with a spectrophotometer [39, 40].

The CIE *L* * *C* * h* system is somewhat more practical than the CIE *L* * *a* * b* system, but many studies also use the CIE *L* * *a* * b* system for color measurements. This does not represent a major difference since the values can be converted to each other without any problems. Under the CIE *L* * *a* * b* system, brightening occurs with the increase of the L* parameter and the decrease of redness (lower a* parameter) and yellowness (lower b* parameter) [41]. In contrast, the application of the CIE *L* * a * b* system is rarely used in clinical practice and is usually used for objective assessment of bleaching quality [41, 42].

For some time, research has been conducted in this field to determine whether it is possible to accelerate the bleaching process with the aid of light sources. The results vary and depend on several factors, such as the bleaching gel or the type of light and their compatibility with each other.

The purpose of light activation is to heat the gel or to heat the pigments of the gel selectively and thus accelerate radical formation, which provides the whitening effect [43]. However, the heating can also transfer to the pulpal tissue and cause irreversible damage [44–46]. The laser parameters used here are based on studies in which no temperatures harmful to the tooth were measured [32, 47]. This explains the lower color whitening compared to comparative studies whose bleaching protocols applied higher energy to the gel and tooth, possibly compromising tooth vitality [25].

According to Hein and co-workers, different lamps in the wavelength range 360–580 nm do not show better bleaching results than without light activation [48].

In contrast, however, Luk and colleagues and Sulieman and co-workers found that activation of bleaching gel with different types of light sources, such as halogen (400–500 nm), infrared (2000–4000 nm) or plasma lamps (400–550 nm), and different laser systems, such as argon ion laser (488 nm), diode laser (830 nm), and CO_2_ laser (10.6 µm), are more effective than conventional bleaching [14, 49].

In contrast to other studies, the naturally given tooth color was used here and the tooth was not artificially colored by incorporation in cola, tea, or coffee [25]. This is also one of the reasons why lower whitening effects were achieved. In contrast to this study, the 940 nm laser resulted in a whitening of ∆E * *ab* = 28.5896 with artificial discolorations. This is because the lighter a tooth is anyway, the smaller will be the anticipated color difference as a result of bleaching [38].

In this case, the shade determination was performed immediately after the bleaching procedure to present immediate results, as is usually shown to the patient in the dental practice while in clinics it is known that the teeth get darker soon.

In other studies, color determination is performed 7 days after bleaching to avoid miss interpretation due to the desiccation effect. In fact, teeth also become whiter when water is isolated [15, 50–52]. However, this can be neglected to a small extent, since in this experimental setup water was continuously pumped through the teeth to simulate physiological blood circulation.

Therefore, a whitening effect solely due to the bleaching process as such can be assumed here. All groups have led to a change in tooth color in the form of an altered ∆*E* * *ab* value. However, this does not necessarily mean that the change is also perceptible to the human eye. ∆*E* * *ab* values less than 3.3 are considered clinically insignificant [53] and about 50% of the population can perceive a color difference of ∆*E* * *ab* = 1 [54]. Another study shows that ∆*E* * *ab* values greater than 2 are detected as color difference in 100% of cases. 2 > ∆*E* * *ab* > 1 > are not always detected and for ∆*E* * *ab* < 1 most errors occur [55]. Based on these values, it can be said that all laser groups resulted in a visually perceptible color difference of the teeth (group1 ∆*E* * *ab* = 4.6, group2 ∆E * *ab* = 3.1, group 3 ∆*E* * *ab* 3.1, Group4 ∆*E* * *ab* = 3.2), whereas the control group without light activation did not (group 5 ∆*E* * *ab* = 0.7).

In this study, LaserWhite20 was used in conjunction with Ezlase 940 nm; this bleaching gel was specially developed for use with the Biolase diode laser system at a wavelength of 940 nm. For the Sirolaser Blue 445 nm and 970 nm, Voco's Perfect bleach + was used. One of the most important factors in bleaching is the H_2_O_2_ concentration, which is the same here in the study for both gels used (35%) [13]. Therefore, the bleaching gel does not form a variable here. The laser and the gel are to be considered as a unit.

In this study, the laser-activated groups (groups 1–4) achieved significantly better bleaching results than the control group (group 5). However, in a study by Strobl et al. and Hahn et al., conventional bleaching achieved better results than laser bleaching [56, 57]. The differences may be because the laser and laser parameters must be matched to the bleaching gel and the desired effect. A red bleaching gel containing 35% H_2_O_2_ with an irradiation time of 30 s was also used by Strobl and co-workers. However, the wavelength of the Nd:YAG laser is 1.064 µm and thus has a lower absorption in the red pigment than the 445 nm laser. At a power of 4W, the bleaching protocol has an eightfold higher power than the bleaching protocol with the 445 nm laser of this study and still achieves worse results. These results give reason to reconsider the laser parameters and to question the use of Nd:YAG laser radiation for in-office bleaching. The diode laser groups 940 nm and 970 nm differ only slightly. This is also observed in other studies [25, 40, 47]. Activation of the bleaching agent may accelerate the bleaching process, although the final result may be similar to conventional bleaching [58].

In principle, however, it is always difficult to compare the results of studies on this topic because they often differed in bleaching agent, bleaching method, and bleaching protocol. For this reason, in this in vitro study, the comparisons among groups should be viewed critically, because the contact time of the gel, the energy dose and the bleaching gel vary from group to group. Nevertheless, it can be concluded that group 1 (diode laser 445 nm) with the second lowest absorbed dose of 53.05 J/cm^2^ leads to the greatest teeth whitening effect. Groups 2 (diode laser 970 nm) and 3 (diode laser 940 nm handpiece) have approximately the same absorbed dose of 106.10 and 105 J/cm^2^ in their whitening protocol, respectively, and both produce results similar to each other but inferior to group 1. Group 4, with the lowest absorbed dose of 47.16 J/cm^2^, leads to results comparable to those of groups 2 and 3 making group 4 relatively more effective. So the 445 nm performs the best which is mainly explained by the increased absorption of the 445 nm wavelength in the red pigment [59, 60]. Since Voco's bleaching gel is colored red, most of the energy is absorbed in the gel, making radical formation and thus the bleaching process more effective than in the comparison groups.

Basically, the results of the ∆*E* * *ab* values and the Vita Shade scores support each other, since in both cases the laser-activated bleaching processes were basically more effective than the control group and, moreover, the diode laser 445 nm performed best. However, it is not negligible that both measured values come from the same device.

In the context of this study, the VITA classical color scale was only intended to serve as a visualization of the measured color differences, since the CIE L * a * *b* system can precisely define a color in space but is not used in dental practice. One problem that arises, however, is that the color patterns, as in almost all color scales, are distributed randomly in color space and do not provide proportional color distances [61]. This fact explains why group 3 (diode laser 940 nm handpiece) was more effective in whitening the teeth in terms of VITA Shades than the ∆*E* * *ab* values expressed. Since the color patterns do not have uniform distances in space, in some cases small color distances in the color space can lead to a jump to the next Vita-Shade. The example images of the teeth before bleaching and after bleaching are to be evaluated in the same way.

The 445 nm diode laser was not investigated at all regarding its whitening effect. Under these experimental conditions, the 445 nm diode laser achieved significantly better whitening effects related to the Delta E value than all other groups (*p* < 0.05).

A limitation of this study is that only the immediate bleaching results were evaluated, which do not provide information on the long-term stability of the whitening. Future studies with longer follow-up are needed to evaluate the long-term outcome of different bleaching protocols. In addition, clinical studies are needed to confirm the in vitro results.

## Conclusion

With these results, it can be concluded that under the conditions of this study, laser-activated bleaching is more effective than conventional bleaching without light activation. The diode laser 445 nm performs best in this study.

## Figures and Tables

**Fig. 4:**
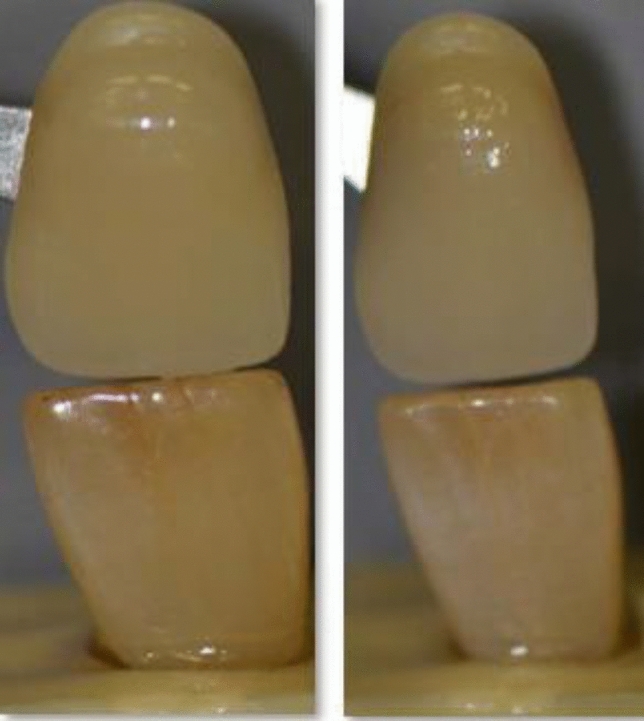
Left: before C2; right: after D3

**Fig. 5:**
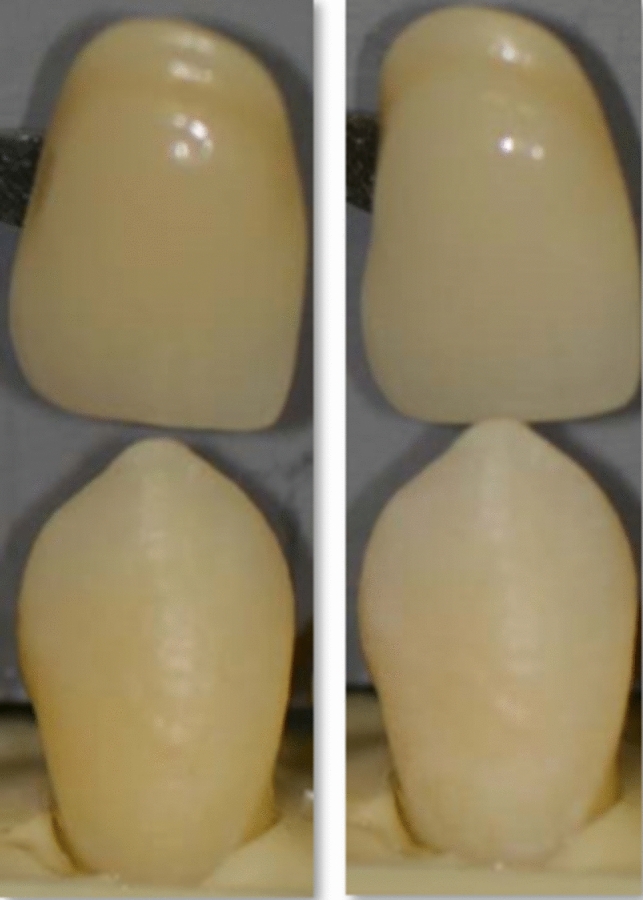
Left: before A3; right: after A2

**Fig. 6:**
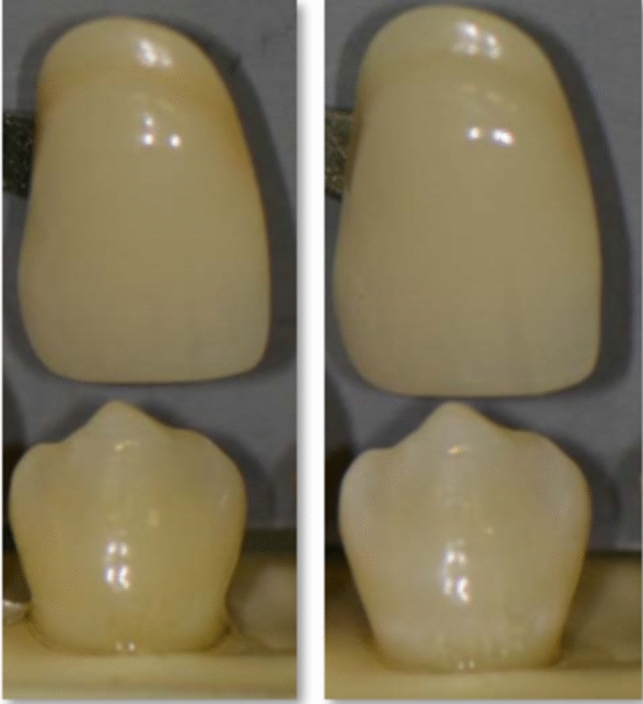
Left: beforeA2; right: After B2

**Fig. 7:**
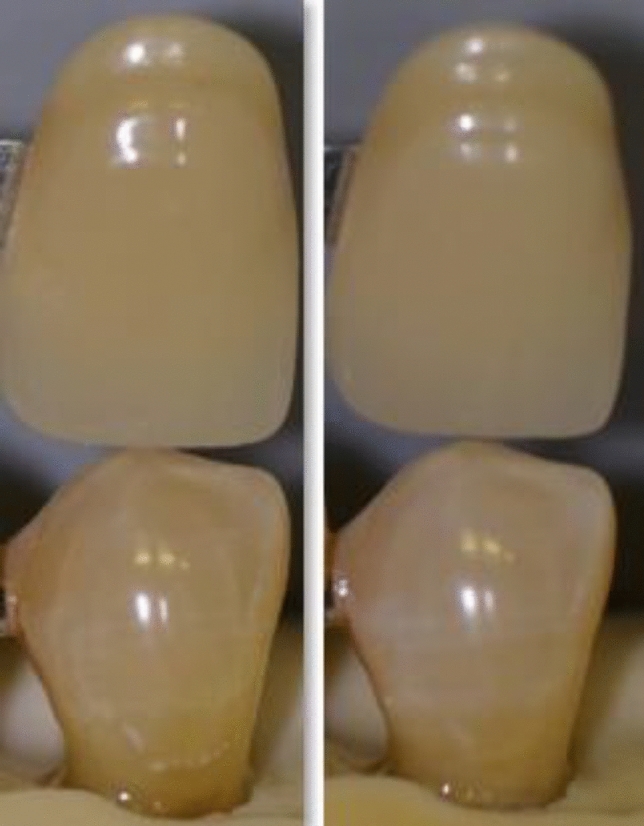
Left: before A3.5; right: after A3

**Fig. 8:**
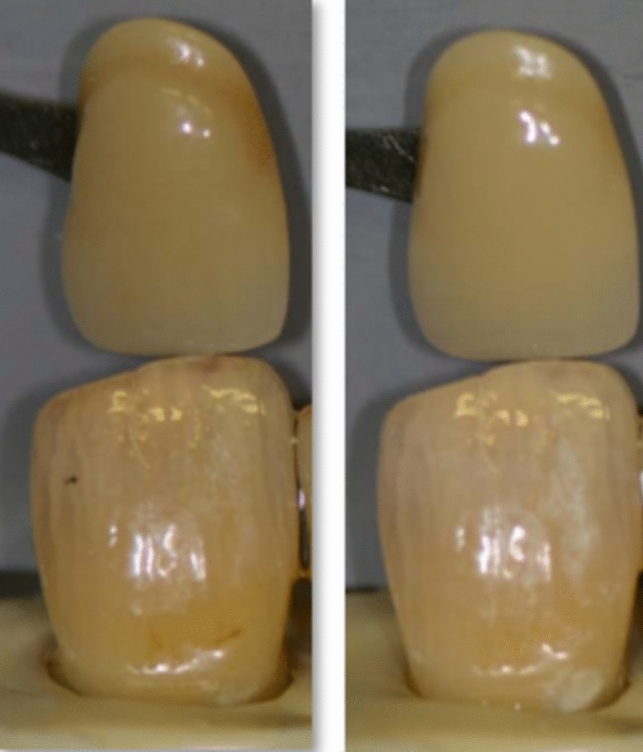
Left: before A4; right: after A3.5

**Fig. 9:**
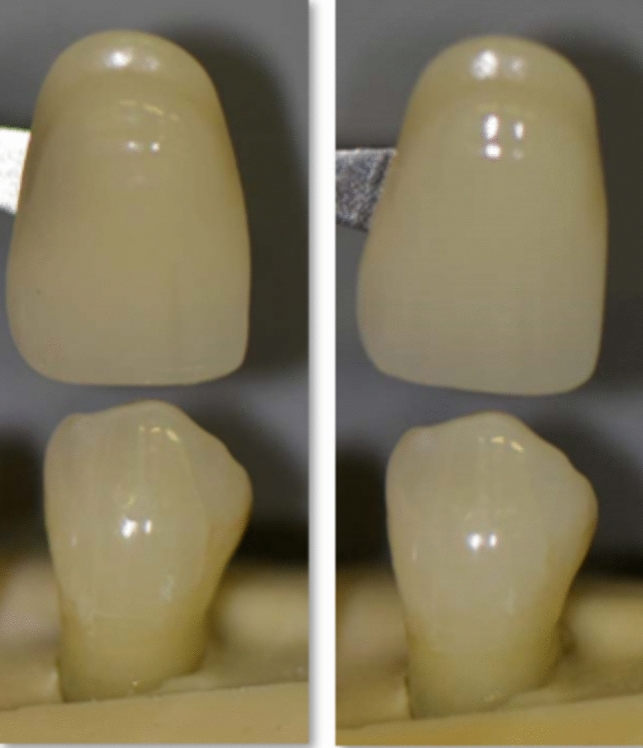
Left: before B2; right: after A1

## Data Availability

The datasets used and analyzed during the current study are available from the corresponding author on reasonable request.

## Appendix

### Example images of the average whitening values of the respective groups

#### Group 1 Sirolaser Blue 445 nm: 4.2 VITA Shades on average (4 rounded down)

See Figs. 4 and 5.

#### Group 2 Sirolaser Blue 970 nm: 1.8 VITA Shades on average (2 rounded up)

See Fig. 6.

#### Group 3 Biolase 940 nm handpiece: 3.4 VITA Shades on average (3 rounded down)

See Figs. 7 and 8.

#### Group 4 Biolase 940 nm fiber: 1.3 VITA Shades on average (1 rounded down)

See Fig. 9.
